# A Novel Plug-Based Vascular Closure Device for Percutaneous Femoral Artery Closure in Patients Undergoing Minimally-Invasive Valve Surgery

**DOI:** 10.3389/fcvm.2021.682321

**Published:** 2021-07-21

**Authors:** Andreas Schaefer, Harun Sarwari, Hermann Reichenspurner, Lenard Conradi

**Affiliations:** Department of Cardiovascular Surgery, University Heart and Vascular Center Hamburg, Hamburg, Germany

**Keywords:** mitral valve, tricuspid valve, minimally invasive cardiac surgery, cardiopulmonary bypass, vascular closure device

## Abstract

**Objectives:** Surgical exposure of groin vessels to establish cardiopulmonary bypass (CPB) for minimally-invasive valve surgery (MIS) is standard of care but may result in postoperative wound healing disorders or seroma formation. Therefore, adaption of transcatheter techniques for fully percutaneous insertion of CPB cannulae may improve clinical results. We herein analyze a single center experience with a novel plug-based vascular closure device for MIS.

**Methods:** Between 03/2020 and 02/2021 MIS using the MANTA™ (Teleflex Medical Inc., Wayne, PA, USA) vascular closure device was performed in 28 consecutive patients (58.8 ± 10.6 years, 60.3% male, logEuroSCORE II 1.1 ± 0.8%) receiving mitral and/or tricuspid valve repair/replacement. Concomitant procedures were left atrial appendage occlusion and cryoablation for atrial fibrillation in 21.4% (6/28) and 10.7% (3/28) of patients, respectively. Data were retrospectively analyzed in accordance with standardized M-VARC definitions. MANTA™ device success and early safety was defined as absence of any access site or access related vascular injury and major and life-threatening bleeding complications.

**Results:** MANTA™ device success with immediate hemostasis and early safety were 96.4% (27/28). In one case, device failure necessitated surgical cut down without further complications. Mean aortic cross clamp time and cardiopulmonary bypass were 96.5 ± 24.2 min and 150.2 ± 33.6 min. Stroke, renal failure or myocardial infarction were not observed. Intensive care unit and total hospital stay were 1.7 ± 0.8 days and 10.1 ± 5.7 days. Overall 30-day mortality was 0%. Post-procedure echocardiography presented one case of residual moderate tricuspid regurgitation and competent valves in all other cases.

**Conclusions:** The MANTA™ device is safe and effective in MIS. Its ease of use and effectiveness to achieve immediate hemostasis have further simplified MIS.

## Introduction

Surgical mitral valve (MV) repair is the gold standard for treatment of primary, degenerative mitral regurgitation (MR). There is a reliable data basis demonstrating efficacy of the surgical approach for this subset of patients also in the long-term (1). Also, complex pathologies, e.g., Morbus Barlow with prolapse of anterior and posterior mitral leaflets, are addressable by surgical annuloplasty with concomitant techniques of leaflet reconstruction (insertion of Neochordae, partial resection of the posterior mitral leaflet) with a high degree of safety, efficacy and excellent results up to 20 years (2). In Germany, 55% of all isolated surgical MV procedures are performed in minimally-invasive, endoscopic (MIS) techniques via right anterolateral minithoracotomy, omitting median sternotomy (3). Also, the tricuspid valve (TV) is amenable by MIS techniques with adequate results for common modes of valve failure. MIS TV repair is feasible in a beating-heart fashion and frequently performed as concomitant procedure in MIS (4, 5).

Commonly, in MIS MV and TV surgery arterial and venous cannulae for cardio-pulmonary bypass (CPB) are inserted via groin vessels by surgical cutdown. However, the surgical coutdown approach inherits a certain risk of postoperative wound healing disorders or seroma formation (6). To reduce possible groin complications, adaption of transcatheter techniques for fully percutaneous insertion of cannulae for establishment of CPB have been described. Established devices for percutaneous vessel closure in MIS valve surgery are ProStar™ XL 10-F system and ProGlide™ devices (both: Abbott Vascular Inc., Abbott Park, IL, USA) (7, 8). Recently, a novel vascular closure device with resorbable collagen material and absence of suture material was introduced for transfemoral transcatheter aortic valve implantation (TAVI). The MANTA™ device (Teleflex Medical Inc., Wayne, PA, US) is adequate for closure of arterial access sites of up to 25 Fr. Safety and efficacy of the system was demonstrated in a real world TAVI patient cohort (9). To date, MIS MV or TV surgery utilizing the MANTA™ system for closure of the femoral artery has only been reported anecdotally (10). We herein report our single center experience with fully percutaneous plug-based vascular closure for cannulation of groin vessels in MIS MV and TV surgery.

## Materials and Methods

### Patients

Between 03/2020 and 02/2021, 28 patients (58.8 ± 10.6 years, 39.7% female, EuroSCORE II 1.1 ± 0.8%) received MIS MV or TV surgery for treatment of mitral and/or tricuspid regurgitation with intraoperative utilization of a novel collagen-based vascular closure device for cannulation of the femoral artery (overall MIS MV and TV surgery volume: 150 per year). Allocation of patients to MV or TV surgery followed current international recommendations (11) after consensus of the local dedicated heart team. The study was approved by the local institutional review board.

### Diagnostic Work-Up and Study Procedure

The pre-procedural diagnostic work-up and the surgical procedure followed institutional standards and were described before (12): all patients received preoperative TTE and TEE for evaluation of cardiac functional status and valve assessment. Furthermore, ultrasound of groin vessels was performed to determine possible stenosis or calcifications at the femoral level. No patient had to be excluded due to stenosis or calcifications seen in preoperative ultrasound. Contrary to TAVI procedures, in this young patient cohort no preoperative computed tomography of iliac vessels or intraoperative ultrasound-guided vessel puncture was performed.

MIS MV and TV surgery was performed under general anesthesia in supine position. Access was gained via a 3–4 cm incision in the right inframammary fold or along the perimammilary margin through the fourth intercostal space. Visualization of the operative field was achieved using a soft-tissue retractor. Three small incisions for insertion of a 3D-HD camera (Aesculap Einstein Vision, Tuttlingen, Germany), a transthoracic aortic clamp and a left atrial retractor were placed. For peripheral cardiopulmonary perfusion, all cannulas were positioned under TEE guidance. A transthoracic Chitwood aortic clamp was used for cross-clamping of the aorta, the heart was arrested with antegrade Del-Nido cardioplegia and moderate hypothermia of 32° was established. After opening of the left/right atrium a dynamic retractor was used to lift the left/right atrial roof. MV/TV surgery techniques included correction of prolapsed anterior and posterior leaflets by chordal replacement, ring annuloplasty and valve replacement.

**Figure 1:**
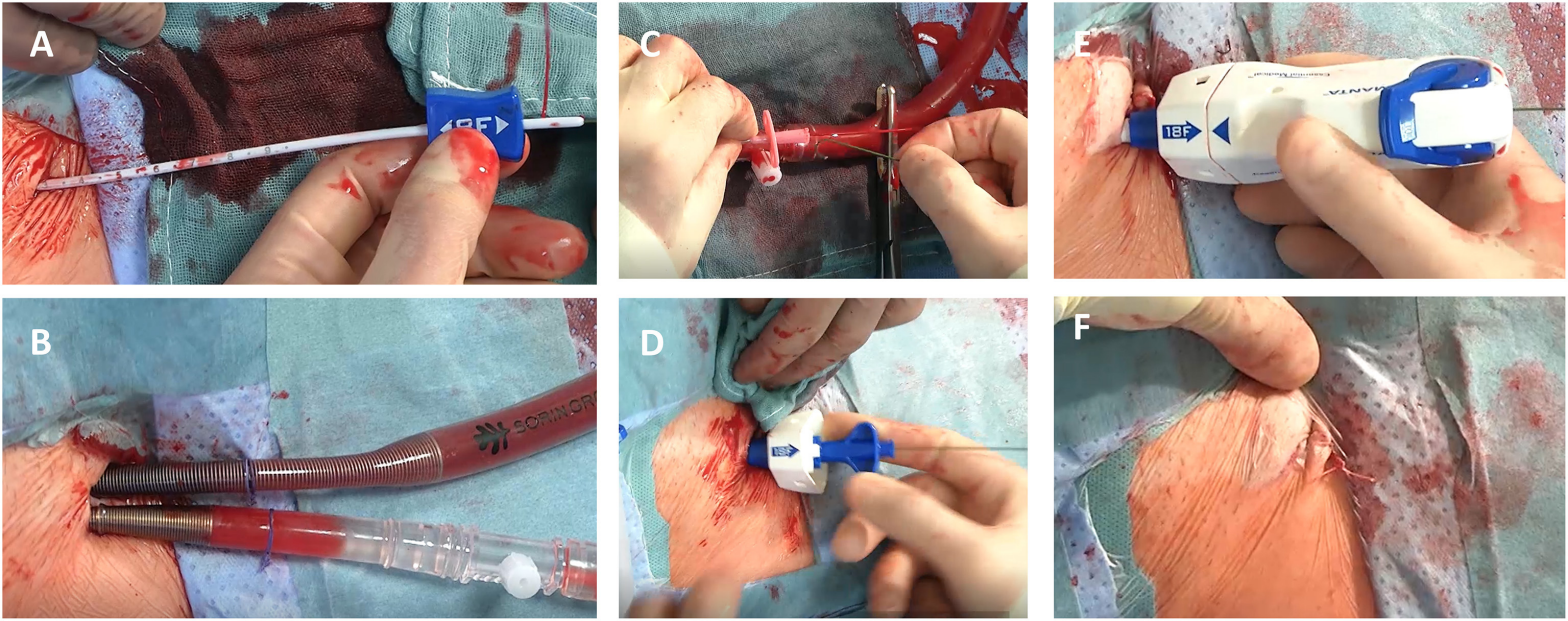
MANTA™ (Teleflex Medical Inc., Wayne, PA, US) vascular closure device for minimally-invasive valve surgery. **(A–F)** demonstrates percutaneous cannulation/decannulation of femoral artery and vein for cardiopulmonary bypass using a fully percutaneous plug-based vascular closure device: In contrast to surgical exposure of groin vessels a depth locator is inserted over the guidewire to determine the distance from skin level to the endoluminal space of the vessel for later device positioning **(A)**. Following this, cardiopulmonary bypass is established for minimally- invasive valve surgery **(B)**. Before final decannulation the arterial cannula is punctured to reinsert the guidewire for percutaneous plug-based vascular closure device **(C)**. Afterwards the MANTA™ sheath **(D)** and the closure unit **(E)** are placed to the predetermined deployment level. After successful hemostasis the MANTA ™ device is removed and the suture is cut at the skin level **(F)**.

For closure of the femoral artery the 18 Fr. MANTA™ device was utilized as previously described (13): depth of the initial puncture was determined using a puncture locating dilator, which was inserted over the wire and detected puncture depth by outlet backflow/ stop of backflow, a principle known from smaller vessel closure devices like the Angio-Seal (Terumo Interventional Systems, Somerset, NJ, US). Depth was defined at skin-level. For later vessel closure device position deployment depth was defined as puncture depth at skin level plus 1 cm. After completion of MIS MV/TV surgery, the arterial cannula was clamped, punctured, a wire was inserted, and the cannula was retracted over the wire. Then, the MANTA™ sheath was fully inserted over the wire and the dilator was removed. Subsequently, the MANTA™ device was inserted over an integrated insertion tool and the whole system was slowly removed in a steady 45° angle under constant retraction force. By observing markings on the sheath, deployment depth was adjusted and a lever was rotated for toggle release. Now, the system was further retracted from the femoral artery until tension appeared and the indicator field showed yellow/green. By advancing the lock advancement tool until a click was heard, the puncture was sealed *via* a collagen plug. The collagen plug is low-profile, non-thrombogenic and bio-resorbable. When hemostatis was obtained the guide-wire was removed, the lead-suture was cut and skin was closed by a single suture (Figure 1). Femoral vein hemostasis was achieved by a deep Prolene suture and consecutive application of pressure after removal of the cannula.

### Statistics

Baseline and intraprocedural data were retrospectively collected and entered into a standardized database and analyzed. Clinical endpoints regarding MV surgery and access site complications and bleeding were adjudicated in accordance with the updated standardized M-VARC definitions (Clinical endpoints: mortality, hospitalization, neurological events, myocardial infarction, access and vascular complications, bleeding complications, acute kidney injury, arrhythmia, device/procedural success) (14). 30-day follow-up was accomplished in all patients. Duplex sonography of iliac vessels was performed in all patients 30 days after the index procedure. Data are presented as absolute numbers and percentages for categorical variables and mean values and standard deviation for continuous variables.

## Results

### Baseline Demographics

The patient cohort presented with a low risk profile for cardiac surgery as determined by common risk stratification tools (STS PROM Score 1.0 ± 0.9%, log EuroSCORE II 1.1 ± 0.8%). A high proportion of patients were highly symptomatic (NYHA ≥ III 19/28, 67.8%) and the entire cohort presented with a low comorbid burden. Most common accompanying diseases were arterial hypertension (9/28, 32.1%) and atrial fibrillation (6/28, 21.4%). Detailed patient demographics are summarized in Table 1.

**Table 1:** Baseline demographics.

	**Study group (*n* = 28)**
Age, years	58.8 ± 10.6
Female gender, % (*n*)	39.7 (10)
BMI, kg/m^2^	24.8 ± 3.9
logEuroSCORE II, %	1.1 ± 0.8
STS PROM Score, %	1.0 ± 0.9
Diabetes mellitus, % (*n*)	0.0 (0)
Arterial hypertension, % (*n*)	32.1 (9)
Pulmonary hypertension, % (*n*)	14.3 (4)
Hyperlipidemia, % (*n*)	25.0 (7)
Stroke, % (*n*)	10.7 (3)
Coronary artery disease, % (*n*)	14.3 (4)
Extracardiac atheropathy^∞^, % (*n*)	4.0 (1)
Arrhythmia, % (*n*)	21.4 (6)
COPD > Gold II^∞^, % (*n*)	0.0 (0)
Creatinine, mg/dl	0.9 ± 0.2
NYHA ≥ III, % (*n*)	67.8 (19)

*BM, Body mass index; logEuroSCORE, logistic European System for Cardiac Operative Risk Evaluation; STS-PROM, Society of Thoracic Surgeons Predicted Risk of Mortality; COPD, chronic obstructive pulmonary disease; NYHA, New York Heart Association functional class*.

∞ *extracardiac atheropathy; ∞COPD according to EuroSCORE definitions*.

### Periprocedural Data

All patients underwent MIS MV/TV surgery due to significant regurgitation *via* right anterolateral minithoracotomy. Valve failure mechanisms included primary, degenerative MR (23/28, 82.1%), secondary, functional MR (1/28, 3.5%) and endocarditis (2/28, 7.1%). Concomitant procedures in MIS MV surgery were tricuspid valve repair (4/28, 14.3%), ablation for AF (3/28, 10.7%) and left atrial appendage closure (6/28, 21.4%). In two patients MV replacement was necessary (7.1%). Isolated TV repair was performed in 7.1% of patients (2/28). For cannulation of the femoral artery most frequently 19 Fr. cannulae were utilized (18/28, 64.3%) followed by 21 Fr. cannulae (9/28, 32.2) and a 17 Fr. cannula in one case. Isolated femoral vein cannulation was common with 67.8% (19/28). In nine patients additional venous drainage was established *via* the jugular vein (9/28, 32.2%). MANTA device success with immediate hemostasis was achieved in 27/28 cases (96.4%). Absence of device success was documented in one case with intraprocedural user error during toggle release and consecutive pull-through of the collagen plug. After surgical cutdown and application of a Prolene purse string suture adequate hemostatis was present.

Aortic cross clamp time and CPB time were 96.5 ± 24.2 min and 150.2 ± 33.6, respectively. Detailed periprocedural data are presented in Table 2.

**Table 2:** Periprocedural data.

	**Study group (*n* = 28)**
**Mitral regurgitation**	
Moderate, % (*n*)	7.1 (2)
Severe, % (*n*)	85.7 (24)
**Mitral valve pathology**	
Primary (Degenerative), % (*n*)	82.1 (23)
Secondary (Functional), % (*n*)	3.5 (1)
Endocarditis, % (*n*)	7.1 (2)
Right anterolateral minithoracotomy, % (*n*)	100 (28)
**Cannulation, % (*****n*****)**	
Femoral artery	100 (28)
17 Fr.	3.5 (1)
19 Fr.	64.3 (18)
21 Fr.	32.2 (9)
Femoral vein 25 Fr.	67.8 (19)
Femoral + jugular vein (25 Fr. + 15 Fr.)	21.4 (6)
Femoral + jugular vein (25 Fr. + 17 Fr.)	10.7 (3)
**Valve surgery, concomitant procedures**	
Isolated mitral valve repair	67.8 (19)
Isolated tricuspid valve repair	7.1 (2)
Mitral + tricuspid valve repair	14.3 (4)
Mitral valve replacement	7.1 (2)
Concomitant ablation for AF	10.7 (3)
Concomitant LAA closure	21.4 (6)
MANTA device success, % (*n*)	96.4 (27)
Aortic cross clamp time, min	96.5 ± 24.2
Cardiopulmonary bypass time, min	150.2 ± 33.6

*Fr, French; AF, atrial fibrillation; LAA, left atrial appendage*.

### Clinical and Echocardiographic Outcome Data

During 30-day follow up no death, stroke, myocardial infarction or kidney injury occurred. In one patient postoperative complete atrioventricular block necessitated permanent pacemaker implantation and one patient presented with wound healing disorder of the thoracotomy. No major bleeding, access site complication or access site related transfusion was documented. Echocardiographic follow-up showed competent MV without residual MR in all MV cases. One patient presented with residual moderate TV regurgitation. Intensive care unit and hospital stay were 1.8 ± 0.9 and 10.9 ± 6.1 days, respectively. For detailed clinical and MANTA device outcome parameter see Tables 3, 4.

**Table 3:** Clinical outcome and echocardiographic results at 30 days.

	**Study group (*n* = 28)**
All-cause mortality (30 days), % (*n*)	0.0 (0)
Stroke, % (*n*)	0.0 (0)
Myocardial infarction, % (*n*)	0.0 (0)
Renal failure (AKIN^*^2,3), % (*n*)	0.0 (0)
PPM implantation, % (*n*)	3.6 (1)
Wound healing disorder (thoracotomy), % (*n*)	3.6 (1)
Residual MR > trace, % (*n*)	0 (0)
Residual TR > mild, % (*n*)	3.6 (1)
Intensive care unit stay, days	1.7 ± 0.8
Hospital stay, days	10.1 ± 5.7

* *AKI, Acute Kidney Injury Network; PP, Permanent pacemaker; MR, mitral regurgitation; TR, tricuspid regurgitation*.

**Table 4:** Vascular closure device outcome parameter at 30 days.

	**Study group (*n* = 28)**
Technical success, % (*n*)	96.4 (27)
Pseudoaneurysm, % (*n*)	0.0 (0)
Dissection, % (*n*)	0.0 (0)
Perforation, % (*n*)	0.0 (0)
Occlusion, % (*n*)	0.0 (0)
Arteriovenous fistula, % (*n*)	0.0 (0)
Hematoma, % (*n*)	0.0 (0)
Conversion to cutdown, % (*n*)	96.4 (27)
Wound healing disorder, % (*n*)	0.0 (0)
Nerve injury, % (*n*)	0.0 (0)
Lymph fistula, % (*n*)	0.0 (0)
Bleeding, major/life threatening, % (*n*)	0.0 (0)
Access site complications, % (*n*)	0.0 (0)
Access site related transfusion, % (*n*)	0.0 (0)

## Discussion

### Main Findings

Main findings of the herein conducted series using the MANTA™ device in MIS valve surgery are (I) the utilized device is safe and effective for closure of the femoral artery with immediate hemostatis in MIS valve surgery, (II) the closure device is simple, offers ease of use and a rapid learning process, and (III) has therefore the potential to further simplify MIS valve surgery and reduce groin wound healing disorders/ seroma formation and hospital stay time.

Application of transcatheter techniques in cardiac surgery procedures and especially MIS valve surgery has gained widespread acceptance over the last years. Examples for this development are establishment of rapid deployment valves, an adaption of the transcatheter heart valve technique for open heart surgery, in clinical daily routine or application of cerebral protection devices in cardiac surgery for patients at particular risk for periprocedural stroke (15, 16). One of the most frequently used transcatheter technique in cardiac surgery is application of vascular closure devices for arterial CPB cannulation, especially in MIS valve surgery to avoid surgical cutdown and exposure of groin vessels. Hereby, commonly utilized closure devices are the ProStar and ProGlide systems. In a series of 300 patients who underwent MIS MV surgery with application of the ProStar device for arterial CPB cannulation no postoperative bleeding complications but 5 cases (1.6%) of bleeding events during introduction of the closure device, two cases (0.6%) of retroperitoneal bleeding and six cases (2.0%) of bleeding events necessitating surgical cutdown were described. Device success was 80.0% in the first 50 patients and 98.8% thereafter, suggesting a steep learning curve. Similar results for the ProStar device are reported by Vergnat and colleagues (7, 17). For the ProGlide device only small series of application in MIS valve surgery are documented (18). However, it is known from a broad adaption in endovascular aortic procedures that device success ranges between 92 and 95% and complication rates are low (19, 20).

In our study a device success rate of 96.4% was documented for the herein utilized closure system due to one pull-through of the collagen plug, most likely to false recall of puncture depth. This emphasizes crucial importance of rigorous documentation of initial puncture depth to avoid device failure. In case of uncertainty regarding toggle release depth, *de-novo* determination of puncture depth can be achieved by advancing the puncture locating dilator again over the wire after puncture and removal of the arterial cannula. On the other hand, when device failure occurs, surgical cutdown can be performed in a controlled manner by compression of the femoral artery proximal to the puncture site. The device success rate in this early series is comparatively high and suggests ease of application, although operators were familiar with the MANTA™ system from TAVI procedures. Further potential advantages of the herein used closure device are absence of suture material and the remaining extravascular stainless steel lock indicating the exact position of vessel entry for possible re-interventions in case of later vessel complications. An earlier study suggested a higher rate of VARC-2 adjudicated vascular complications with the MANTA device compared to surgical cutdown (21). However, in this series no postoperative vessel complications in terms of bleeding, wound healing disorders/seroma formation or pseudoaneurysms were seen during 30-days follow-up, which was also seen in a previous study (22) and which further corroborates the assumption that the MANTA™ device has the potential to simplify MIS valve surgery and to reduce intra- and postoperative vessel complications. It has to be emphasized that results of the MANTA device in TAVI differ significantly, most likely due to a significant older patient population with small and/or calcified iliac vessels (23, 24). These results will have to be confirmed in larger patient numbers for further clinical evaluation.

### Study Limitations

Limitations are inherent in a single-center study design with limited patient numbers: patients were not randomized to a specific treatment or vessel closure device, therefore patient preselection with hidden confounders may apply. Furthermore, the herein treated young patient cohort presented non-calcified vessels, facilitating vessel closure with the herein described device. Clinical results may differ in small and/or calcified vessels as known from TAVI procedures.

## Conclusions

The MANTA™ device is safe and effective in minimally-invasive valve surgery. Its ease of use and effectiveness to achieve immediate hemostasis have further simplified minimally-invasive valve surgery at our center.

## Data Availability Statement

The raw data supporting the conclusions of this article will be made available by the authors, without undue reservation.

## Ethics Statement

Ethical review and approval was not required for the study on human participants in accordance with the local legislation and institutional requirements. Written informed consent for participation was not required for this study in accordance with the national legislation and the institutional requirements.

## Author Contributions

AS made substantial contributions in data collection, study design, and writing the manuscript. HS made substantial contributions in data collection, statistical computation, and revising of the manuscript. HR made substantial contributions in study design and revising of the manuscript. LC made substantial contributions in data collection, study design, and writing and revising of the manuscript. All authors contributed to the article and approved the submitted version.

## Conflict of Interest

The authors declare that the research was conducted in the absence of any commercial or financial relationships that could be construed as a potential conflict of interest.

## Abbreviations

CPB: Cardiopulmonary bypass
MIS: Minimally-invasive cardiac surgery
MR: Mitral regurgitation
MV: Mitral valve
TAVI: Transcatheter aortic valve implantation
TV: Tricuspid valve.

## References

[1] David TE Ivanov J Armstrong S Rakowski H. Late outcomes of mitral valve repair for floppy valves: Implications for asymptomatic patients. J Thorac Cardiovasc Surg. (2003) 125:1143–52. 10.1067/mtc.2003.406 12771888

[2] David TE David CM Lafreniere-Roula M Manlhiot C. Long-term outcomes of chordal replacement with expanded polytetrafluoroethylene sutures to repair mitral leaflet prolapse. J Thorac Cardiovasc Surg. (2020) 160:385–94. 10.1016/j.jtcvs.2019.08.006 31570218

[3] Beckmann A Meyer R Lewandowski J Markewitz A Harringer W. German heart surgery report 2018: the annual updated registry of the German Society for Thoracic and Cardiovascular Surgery. Thorac Cardiovasc Surg. (2019) 67:331–44. 10.1055/s-0039-1693022 31311036

[4] Abdelbar A Niranjan G Tynnson C Saravanan P Knowles A Laskawski G. Endoscopic tricuspid valve surgery is a safe and effective option. Innovations (Phila). (2020) 15:66–73. 10.1177/1556984519887946 31903869

[5] Pfannmueller B Misfeld M Davierwala P Weiss S Borger MA. Concomitant tricuspid valve repair during minimally invasive mitral valve repair. Thorac Cardiovasc Surg. (2020) 68:486–91. 10.1055/s-0039-1700506 31891950

[6] Ko K de Kroon TL Post MC Kelder JC Schut KF Saouti N. Minimally invasive mitral valve surgery: a systematic safety analysis. Open Heart. (2020) 7:e001393. 10.1136/openhrt-2020-001393 33046594 PMC7552840

[7] Pozzi M Henaine R Grinberg D Robin J Saroul C Delannoy B. Total percutaneous femoral vessels cannulation for minimally invasive mitral valve surgery. Ann Cardiothorac Surg. (2013) 2:739–43. 24349975 10.3978/j.issn.2225-319X.2013.08.02 PMC3856992

[8] Kim J Yoo JS. Totally endoscopic mitral valve repair using a three-dimensional endoscope system: initial clinical experience in Korea. J Thorac Dis. (2020) 12:705–11. 10.21037/jtd.2019.12.126 32274136 PMC7138998

[9] Kroon HG Tonino PAL Savontaus M Amoroso G Laine M Christiansen EH. Dedicated plug based closure for large bore access -The MARVEL prospective registry. Catheter Cardiovasc Interv. (2021) 97:1270–8. 10.1002/ccd.29439 33347739 PMC8246962

[10] Van Praet KM Kofler M Jacobs S Falk V Unbehaun A Kempfert J. The MANTA vascular closure device for percutaneous femoral vessel cannulation in minimally invasive surgical mitral valve repair. Innovations (Phila). (2020) 15:568–571. 10.1177/1556984520956300 32993410

[11] Writing Committee Members Otto CM Nishimura RA Bonow RO Carabello BA Erwin JP 3rd. 2020 ACC/AHA guideline for the management of patients with valvular heart disease: A Report of the American College of Cardiology/American Heart Association Joint Committee on Clinical Practice Guidelines. J Am Coll Cardiol. (2020):S0735–1097. 10.1016/j.jacc.2020.11.035 33342587

[12] Westhofen S Conradi L Deuse T Detter C Vettorazzi E Treede H. A matched pairs analysis of non-rib-spreading, fully endoscopic, mini-incision technique versus conventional mini-thoracotomy for mitral valve repair. Eur J Cardiothorac Surg. (2016) 50:1181–7. 10.1093/ejcts/ezw184 27261077

[13] Schaefer A Schirmer J Schofer N Schneeberger Y Deuschl F Blankenberg S. Transaxillary transcatheter aortic valve implantation utilizing a novel vascular closure device with resorbable collagen material: a feasibility study. Clin Res Cardiol. (2019) 108:779–86. 10.1007/s00392-018-1407-z 30560381

[14] Stone GW Adams DH Abraham WT Kappetein AP Généreux P Vranckx P. Mitral Valve Academic Research Consortium (MVARC). Clinical trial design principles and endpoint definitions for transcatheter mitral valve repair and replacement: part 2: endpoint definitions: a consensus document from the mitral valve academic research consortium. J Am Coll Cardiol. (2015) 66:308–21. 10.1093/eurheartj/ehv333 26184623

[15] Ensminger S Fujita B Bauer T Möllmann H Beckmann A Bekeredjian R. GARY executive board. rapid deployment versus conventional bioprosthetic valve replacement for aortic stenosis. J Am Coll Cardiol. (2018) 71:1417–28. 10.1016/j.jacc.2018.01.065 29598861

[16] Capestro F Berretta P Alfonsi J Cefarelli M Pierri M Di Eusanio M. Catheter-based cerebral protection system in open cardiac surgery: An example of true hybrid surgery. Multimed Man Cardiothorac Surg. (2020) 22:2020. 32356620 10.1510/mmcts.2020.012

[17] Vergnat M Finet G Rioufol G Obadia JF. Percutaneous femoral artery access with Prostar device for innovative mitral and aortic interventions. Eur J Cardiothorac Surg. (2011) 39:600–2. 10.1016/j.ejcts.2010.06.022 20739185

[18] Ramponi F Yan TD Vallely MP Wilson MK. Total percutaneous cardiopulmonary bypass with Perclose ProGlide. Interact Cardiovasc Thorac Surg. (2011) 13:86–8. 10.1510/icvts.2010.263715 21482577

[19] Sahin AA Guner A Demir AR Uzun N Onan B Topel C. Comparison between PeRcutanEous and surgical femoral aCcess for endovascuLar aOrtic repair in patientS with typE III aortic Dissection (PRECLOSE Trial). Vascular. (2020) 14:1708538120965310. 10.1177/1708538120965310 33054676

[20] Malkawi AH Hinchliffe RJ Holt PJ Loftus IM Thompson MM. Percutaneous access for endovascular aneurysm repair: a systematic review. Eur J Vasc Endovasc Surg. (2010) 39:676–82. 10.1016/j.ejvs.2010.02.001 20185341

[21] Kastengren M Svenarud P Källner G Settergren M Franco-Cereceda A Dalén M. Percutaneous vascular closure device in minimally invasive mitral valve surgery. Ann Thorac Surg. (2020) 110:85–91. 10.1016/j.athoracsur.2019.10.038 31794742

[22] Ahmad AE Salamate S Amer M Sirat S Monsefi N Bakhtiary F. First experiences with manta vascular closure device in minimally invasive valve surgery. Thorac Cardiovasc Surg. (2020). 10.1055/s-0040-1718773. [Epub ahead of print]. 33225434

[23] Wood DA Krajcer Z Sathananthan J Strickman N Metzger C Fearon W. SAFE MANTA study investigators. pivotal clinical study to evaluate the safety and effectiveness of the manta percutaneous vascular closure device. Circ Cardiovasc Interv. (2019) 12:e007258. 10.1161/CIRCINTERVENTIONS.119.007258 31296082

[24] De Palma R Settergren M Rück A Linder R Saleh N. Impact of percutaneous femoral arteriotomy closure using the MANTA TM device on vascular and bleeding complications after transcatheter aortic valve replacement. Catheter Cardiovasc Interv. (2018) 92:954–61. 10.1002/ccd.27595 29575678

